# Thinking Slow More Quickly: Development of Integrated Illness Scripts to Support Cognitively Integrated Learning and Improve Clinical Decision-Making

**DOI:** 10.1007/s40670-021-01293-z

**Published:** 2021-05-04

**Authors:** Leslie H. Fall, Robin English, Tracy B. Fulton, David M. Harris, Khiet Ngo, James Nixon, Jacqueline Hembrook-Short, Amy Wilson-Delfosse

**Affiliations:** 1 Aquifer, Inc., 21 Lafayette Street, Suite 230, Lebanon, NH 03755 USA; 2 Louisiana State University School of Medicine, Baton Rouge, USA; 3 University of California San Francisco School of Medicine, San Francisco, USA; 4 University of Central Florida School of Medicine, Orlando, USA; 5 Loma Linda University School of Medicine, Loma Linda, USA; 6 University of Minnesota School of Medicine, Minneapolis, USA; 7 Case Western Reserve University School of Medicine, Cleveland, USA

**Keywords:** Illness script, Cognitive integration, Curriculum development, Teaching and learning, Basic science education

## Abstract

Illness scripts describe the mental model used by experienced clinicians to store and recall condition-specific knowledge when making clinical decisions. Studies demonstrate that novice clinicians struggle to develop and apply strong illness scripts. We developed the Integrated Illness Script and Mechanism of Disease (IIS-MOD) map framework to address this challenge.

The term “illness script” describes the mental model by which a clinician organizes, stores, and retrieves from long-term memory key concepts and their relationships to clinical problems (i.e., diseases, conditions, or syndromes) [1]. Experienced clinicians draw from a repertoire of scripts to drive purposeful clinical data gathering, compare and contrast diagnostic hypotheses, and direct initial management decisions [2]. Scripted knowledge of each condition is organized into three main components: Enabling Conditions (predisposing epidemiologic and structural factors that influence a patient’s probability of the disease), Fault (the underlying pathophysiological insult), and Clinical Consequences (the patient’s chief concern, signs, and symptoms to which the Fault gives rise) [1, 2]. Importantly, research demonstrates that how a clinician “encapsulates” relevant information within these components facilitates or hinders accurate and efficient information retrieval during clinical decision-making [1].

The development of a holistic, organized knowledge base and a strong foundation of core illness scripts is a principal goal of health professions training. Initially, clinical learners must rely heavily on basic science understanding of core concepts and underlying pathophysiological mechanisms (e.g., the Fault) to slowly reason through clinical hypotheses and competing diagnoses. Not surprisingly, effective cognitive integration of basic and clinical science knowledge has been shown to enhance diagnostic accuracy of novice clinicians [3, 4]. However, left to their own devices’ learners will make connections, but seldom do they make the correct connections between biomedical knowledge and clinical features [3, 4]. The problem is further compounded by the difficulty basic science and clinical faculty face in “unpacking” their own deeply interconnected and encapsulated knowledge while teaching and collaborating [1].

We developed the model of an Integrated Illness Script (IIS) and related Mechanism of Disease (MOD) map to address these challenges and accelerate the development and effective use of scripts by faculty and learners (Fig. 1). The IIS provides support for inductive reasoning from the observed features back through the relevant mechanisms and basic science concepts to the originating insult. The MOD map, on the other hand, provides a holistic and deductive visual representation of the clinical path: from the original insult, through the causal mechanisms and their corresponding concepts, to the resulting clinical features seen at presentation. The Overview section of the IIS provides a brief clinical definition, a description of the initiating pathophysiological insult, and a concise summary of the most salient basic science concepts impacted by the underlying disease. The Epidemiology section describes risk factors and their underlying mechanisms, highlighting pathology and genetics concepts. The Key Clinical Features articulate the most common presenting clinical findings as well as the basic science causal mechanisms that explain why each feature occurs in the condition. Links to core basic science concepts underlying the mechanisms are included (www.aquifersciences.org). The MOD map flows consistent with the manner a basic scientist would explain the occurrence of a given feature in a known disease and provides an integrated scientific view of the condition. In each section, annotated references are provided to supply supporting evidence for emerging and cutting-edge concepts and mechanisms.

**Fig. 1:**
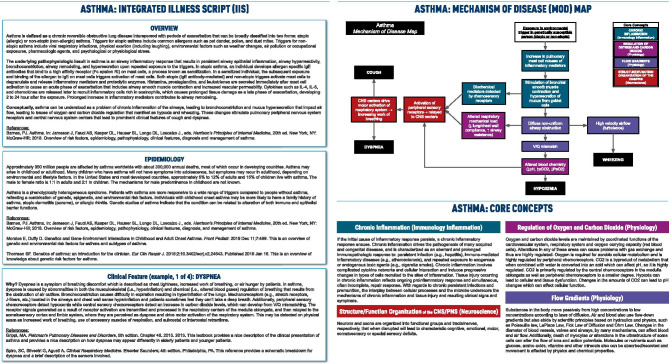
Integrated Illness Script (IIS) and Mechanism of Disease (MOD) map for asthma

The structure of the IIS-MOD map was created through an iterative multidisciplinary and multi-institutional consensus process of leading clinical and basic science educators and validated through multiple national workshops over a 3-year period. The design was advanced by six nationally selected pilot schools with teams of basic science and clinical educators and senior medical students working in rapid cycle prototypes to create content for fifty exemplar IIS-MOD maps. To date, six medical schools are utilizing the IIS and MOD map models as pedagogical tools to educate learners at all levels.

For novice learners, the IIS and MOD map can provide a cognitively integrated framework with which to develop, apply, and elaborate basic science knowledge in a manner that supports emerging clinical decision-making skills. For experienced basic science and clinical faculty, the IIS and MOD map facilitate the difficult task of “unpacking” and making transparent deeply encapsulated knowledge to learners and collaborating colleagues. Importantly, the development and use of the IIS have enabled more effective collaborative instructional design and teaching between scientists and clinicians, and between scientists across disciplines. We believe the use of IIS-MOD maps has the potential to enhance curriculum development, teaching, and learning and to advance the value and safety of patient care by both novice and experienced clinicians.

## References

[1] Schmidt HG, Boshuizen HPA (1993). On acquiring expertise in medicine. Educ Psychol Rev.

[2] Bowen JL (2006). Educational strategies to promote clinical diagnostic reasoning. N Engl J Med.

[3] Baghdady M, Carnahan H, Lan EWN, Woods NN (2013). Integration of basic sciences and clinical sciences in oral radiology education for dental students. J Dent Educ.

[4] Kulasegaram KM, Manzone JC, Ku C, Skye A, Wadey V, Woods NN (2015). Cause and effect: testing a mechanism and method for the cognitive integration of basic science. Acad Med.

